# Social and family factors as determinants of exercise habits in Japanese elementary school children: a cross-sectional study from the Super Shokuiku School Project

**DOI:** 10.1186/s12199-020-00892-3

**Published:** 2020-09-14

**Authors:** Satomi Sawa, Michikazu Sekine, Masaaki Yamada, Yugo Fukazawa, Yusuke Hiraku

**Affiliations:** 1 School of Human Development, University of Toyama, 3190 Gofuku, Toyama, 930-8555 Japan; 2 Department of Epidemiology and Health Policy, School of Medicine, University of Toyama, 2630 Sugitani, Toyama, 930-0194 Japan; 3 Department of Brain Structure and Function, Research Center for Child Mental Development, Faculty of Medical Sciences, University of Fukui, 23-3 Matsuoka-Shimoaizuki, Eiheiji, Yoshida, Fukui, 910-1193 Japan; 4 Department of Environmental Health, University of Fukui School of Medical Sciences, 23-3 Matsuoka-Shimoaizuki, Eiheiji, Yoshida, Fukui, 910-1193 Japan

**Keywords:** Elementary school children, Physical activity, Parental lifestyle, Social background

## Abstract

**Background:**

Many studies have already reported on the relationship between exercise habits and health among schoolchildren. However, few have examined social and/or family factors as determinants of exercise habits.

**Methods:**

This study’s participants included 1721 schoolchildren aged between 6 and 13 who were involved in the Super Shokuiku School Project in January 2016. A survey was conducted to assess gender, grade level, physical activity, lifestyle, overall health, enrichment of school life, social background, and parental lifestyles. Both dislike and lack of physical activity were used to measure poor exercise habits; correlates were analyzed using logistic regression.

**Results:**

“Lack of close friends” had the strongest links with both dislike (adjusted odds ratio [OR] 5.30; 95% confidence interval [CI], 2.78–10.1) and lack of (adjusted OR 5.40; 95% CI, 2.81–10.4) physical activity. Further, children who engaged in long periods of screen time and lacked parental communication also tended to dislike and lack physical activity. Children with mothers who were unemployed (housewives) and had unhealthy lifestyles, as well as those with poor health, were also more likely to lack physical activity.

**Conclusion:**

Social and family factors (e.g., having close friends) may be determinants of exercise habits among schoolchildren, independent of their own lifestyle factors. Although a longitudinal study is needed to determine causality, substantial attention may thus be required to these factors when promoting physical activity in children.

## Background

The Physical and Athletic Aptitude Survey [1] conducted by the Japanese Ministry of Education, Culture, Sports, Science and Technology (MEXT) indicates that there is a widening physical ability gap between children who engage in physical activity and those who do not [2]. Moreover, the number of children who do not exercise increases with age [3]. Such reductions may be associated with lower levels of motivation, emotional strength, and the ability to form interpersonal relationships. As a consequence, lack of physical activity may negatively influence psychosocial development [4]. The Japan Sports Agency has thus proposed a goal of increasing the rate of sports participation from 58.7 to 80% among junior high school students (aged between 13 and 15) while decreasing the percentage of them who dislike sports from 16.4 to 8% by the year 2022 [5]. In this context, it is important to research the reasons for the growing lack of physical activity and sedentary lifestyles among these children to determine what measures are needed to develop good exercise habits during early childhood.

Children are now spending less time playing outside and/or participating in sports. By contrast, they are spending longer hours indoors while watching television and playing video games. Many studies have linked such physical inactivity to overweight and obesity [6], peer problems [7], and school-avoidant emotions [8]. Further, children with overweight or obese mothers tend to engage in less physical activity and to spend more time watching television and playing game console or gaming device [9]. Similarly, children who are in poorer health and have parents that are heavy internet users tend to clock longer screen times [10]. These findings provide evidence that child health is significantly influenced by health-related parental behavior [9–11]. Conversely, studies have reported that even short durations of physical activity effectively reduce anxiety and/or boost self-esteem [7], while consistent physical activity can help mitigate emotional problems [12]. Moreover, elementary schools that successfully reduced rates of child inactivity were also able to increase the number of students who enjoyed physical engagement [13]. This implies that physical activity can be encouraged through enjoyment, thus promoting wholesome physical and mental health while providing fulfilling school experiences.

Meanwhile, Japan has one of the highest rates of child poverty among all Organisation for Economic Co-operation and Development (OECD) nations. In this context in particular, widening socioeconomic disparities and their effects on child health are serious concerns [14, 15]. As such, health education in the school setting should make it possible for both parents and children to learn regardless of academic achievement [14] or socioeconomic status [14–17]. These measures are now highly anticipated. Notably, previous studies have reported that lifestyle is a major influencing factor for children who dislike and lack physical activity [18, 19]. However, few studies have examined how these factors are related to the family environment and other social factors surrounding the child. This study therefore comprehensively investigated the factors related to both dislike of and lack of physical activity among Japanese children, including habits that relate to health and lifestyle, the family environment, and other social factors.

## Methods

### Participants and survey outline

**Fig. 1:**
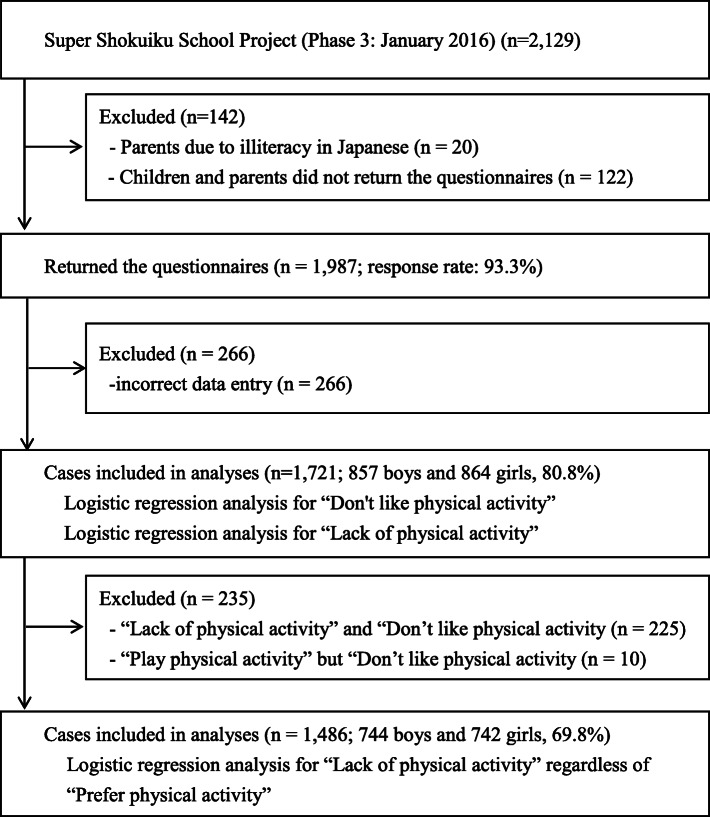
Participants and survey outline

Figure 1 shows the participants and the survey outline. A total of 2129 children aged between 6 and 13 who were involved in the Super Shokuiku School Project (Phase 3: January 2016) [10, 20] and who attended one of five elementary schools in the city of Takaoka, Toyama Prefecture, participated in this study, as did their parents. Twenty children were excluded due to their parents’ illiteracy in Japanese. A total of 1987 children agreed to participate in our survey and returned the questionnaires (response rate 93.3%). The Super Shokuiku School Project was designed to investigate food education and was supported by MEXT. The overall purpose of the project was to promote healthy lifestyles in schoolchildren and improve their health. The survey was approved by the Ethics Committee of the University of Toyama. Written informed consent was obtained from the participants’ parents, and the participants provided assent; participation was voluntary.

### Questionnaire

We used children’s lifestyle and social and family factors cleared for use in our previous cohort studies [9, 19, 21]. Furthermore, we used factors that showed significant differences in previous Super Shokuiku Project studies [8, 10, 11]. Participants were asked to complete questionnaires asking about gender, grade level, physical activity, lifestyle, overall health, enrichment of school life, social background, and parental lifestyles. Participants’ grade levels were classified according to the Japanese curriculum guidelines. Considering the child’s developmental stage, the composition of goals for Japanese physical education curriculum guidelines was divided into three stages: low (1st and 2nd grades), middle (3rd and 4th grades), and high (5th and 6th grades). The children responded, by themselves or with their parents if necessary, to items concerning gender, grade, physical activity, lifestyle, overall health, and enrichment of school life, while their parents responded to items concerning social background and parental lifestyle. Participants returned the completed questionnaires to their schools.

#### Physical activity

The physical activity portions of the questionnaire included items on “frequency of physical activity” [21] and “preference for physical activity” [18]. Responses for “frequency of activity” were answered on a 4-point scale and divided into the two following categories: “Very often (very often, often)” and “Not often (rarely, almost never).” Responses for “preference for physical activity” were also answered according on a 4-point scale, divided into: “Like very much (like very much, like)” or “Dislike (don’t like so much, dislike).” Validity for both these scales was measured by a previous study [21]. In this context, a high frequency of physical activity was significantly associated with an increasing trend in energy expenditure originating from physical activity.

#### Social background and parental lifestyles

Social background was assessed based on the following items: mother’s employment status, family structure, perceived family affluence, communication with parents, individual parental internet use at home (h/day, both mothers and fathers), and parental health behaviors (both mothers and fathers) [10, 11]. The item concerning “mother’s employment status” [20, 22] included three response categories (“full-time,” “part-time,” and “unemployed (housewives)”), while “family structure” [22, 23] was categorized as either “three-generation family” or “nuclear family.” Further, “socioeconomic status” was determined according to perceived “family affluence” [20, 22] (i.e., “affluent,” “not affluent,” or “neither”), while “communication with parents” [22, 24] was categorized as either “often” or “rarely.” We also asked for the total time parents spent using the internet (IU) at home in h/day; here, responses were given according to a 6-point scale and divided into the two categories, “< 2 h (none or almost none, < 1 h, and 1 h to < 2 h)” and “≥ 2 h (2 h to < 3 h and ≥ 3 h).” This was based on a 2018 report indicating that Japanese adults in their 30s spent an average of about 1.5 h per day on the internet [10, 25]. Breslow’s seven good health-related behaviors have been widely accepted for use in developed countries and were thus used as parental health indicators in this study [26]. The behaviors include (1) adequate sleep time, (2) not smoking, (3) appropriate weight control, (4) not drinking excessively, (5) regular physical activity, (6) not skipping breakfast, and (7) not frequently snacking. Parents answered “yes” or “no” to the seven items; “yes” responses were summed to provide cumulative behaviors ranging from 0 to 7, which we categorized into three groups, just as in previous research: “low” (0–3), “middle” (4–5), and “high” (6–7) [10, 11].

#### Child lifestyle factors

Child lifestyles were assessed based on breakfast consumption, nighttime sleep duration, screen time in h/day, and after-school “cram school” attendance. Here, “breakfast consumption” [10, 27] was classified into two categories, “eat every day” and “skipping breakfast,” while “nighttime sleep duration” [23] was categorized according to the number of sleeping hours (i.e., “more than 8 hours” and “less than 8 hours”). This was based on previous research indicating that Japanese elementary schoolchildren average approximately 8.5 h of sleep per night [25]. The question about “screen time in h/day” for children included television and film viewing, gaming, and internet use [20]. Here, responses were given according to a 6-point scale and divided into the two categories of “< 2 h (none, almost none, < 1 h, and < 2 h)” and “≥ 2 h (< 3 h, 3 to < 4 h, and ≥ 4 h).” The Japan Pediatric Association recommends that total screen time be limited to < 2 h per day [25].

#### Overall health among child participants

A question from a validated Japanese version of the Dartmouth Primary Care Cooperative Information Project (COOP) was used to evaluate overall health among children [18, 19, 28]. They were asked the following: “During the past 4 weeks, how would you rate your physical and mental health in general?” Participants who rated their overall health as “excellent,” “very good,” or “good” were classified as having “good health status,” while those who answered “fair” or “poor” were classified as having “poor health status.”

#### Enrichment of school life

We asked two questions to subjectively assess “enrichment of school life,” including “Do you have close friends?” [29] and “Are you able to understand school lessons well?” [20]. Responses were given according to 4- and 5-point scales, respectively, and then divided into the following respective categories for each question: “yes (many, a few)” or “no (not many, no friends)” and “understand well (well, relatively well)” or “do not understand (neither, relatively poor, and poor).”

### Statistical analyses

Taking into consideration gender (boys or girls) differences for children’s lifestyles and physical activity participation, a Chi-square test was performed to compare variables by gender. Correlations and multicollinearity between independent variables were examined using Spearman rank correlation coefficients. Logistic regression analyses were conducted to evaluate the strengths of the associations between child physical activity and the items for social background, parental lifestyles, child lifestyles, child health, and enrichment of school life. All variables were simultaneously entered into the model during multivariate analyses. Odds ratios (ORs) and 95% confidence intervals (CIs) were also calculated. We conducted the Hosmer−Lemeshow test, and sub-group (gender) for which the regression equations showed good fit were used. Finally, logistic regression analyses were performed to determine the strengths of the associations between “lack of physical activity” regardless of “preference for physical activity” and the items for social background, parental lifestyle, child lifestyle, overall child health, and enrichment of school life. All analyses were conducted using the SPSS software version 26.0 J (IBM, Armonk, NY, USA). Two-tailed *P* values less than 0.05 were considered statistically significant for all tests.

## Results

Table 1 shows the results for participant characteristics. Of the 2129 respondents who returned their questionnaires, 1721 (80.8%; 857 boys and 864 girls) answered all relevant items, and thus were included in our analysis. Regarding child physical activity, 13.7% of respondents (*n* = 235) did not like it, while 27.7% (*n* = 476) lacked physical activity. Regarding social background factors, the most frequent answers to the items assessing mothers’ employment status, family structure, and perceived family affluence were “part time,” “nuclear family,” and “neither,” respectively. Regarding parental lifestyles, the most frequent answers to the questions assessing parental internet use at home were “< 2 h,” and for parental (e.g., mother and father) health behaviors [10, 11] according to Breslow’s health score [26] were “father is middle” and “mother is middle,” respectively. There were no significant differences in mother’s employment status, family structure, perceived family affluence, father’s IU at home, father’s health behaviors, mother’s health behaviors, breakfast consumption, sleep duration, preference for physical activity, frequency of physical activity, cram school attendance, overall health, or understanding the school lessons by gender. However, girls were more likely than boys to communicate with their parents and have close friends, while boys were more likely to have ≥ 2 h screen time per day, and their mothers tended to use the internet at home longer per day than girls’ mothers.

**Table 1:** Participant characteristics by gender (*n* = 1721)

Variable		Total		Boys		Girls		*p* value
		*n* = 1721	%	*n* = 857	%	*n* = 864	%	
Grade	Low (1st–2nd)	574	33.4	292	34.1	282	32.6	0.818
	Middle (3rd–4th)	545	31.7	269	31.4	276	31.9	
	High (5th–6th)	602	35.0	296	34.5	306	35.4	
**Social background and parental lifestyles**								
Mother’s employment status	Full time	698	40.6	337	39.3	361	41.8	0.560
	Part time	777	45.1	393	45.9	384	44.4	
	Unemployed (housewives)	246	14.3	127	14.8	119	13.8	
Family structure	3-generation family	575	33.4	268	31.3	307	35.5	0.061
	Nuclear family	1146	66.6	589	68.7	557	27.0	
Perceived family affluence	Affluent	460	26.7	227	26.5	233	49.1	0.218
	Neither	818	47.5	394	46.0	424	24.0	
	No affluent	443	25.7	236	27.5	207	46.7	
Communication with parents	Often	1428	83.0	650	75.8	778	90.0	< 0.001
	Rarely	293	17.0	207	24.2	86	10.0	
Father’s IU at home, h/day	< 2 h	1471	85.5	727	84.8	744	50.6	0.451
	≥ 2 h	250	14.5	130	15.2	120	48.0	
Mother’s IU at home, h/day	< 2 h	1622	94.2	797	93.0	825	95.5	0.027
	≥ 2 h	99	5.8	60	7.0	39	4.5	
Father’s behaviors based on Breslow’s health score	High (6, 7)	365	21.2	187	21.8	178	20.6	0.141
	Middle (4, 5)	726	42.2	376	43.9	350	40.5	
	Low (0–3)	630	36.6	294	34.3	336	38.9	
Mother’s behaviors based on Breslow’s health score	High (6, 7)	460	26.7	234	27.3	226	26.2	0.784
	Middle (4, 5)	932	54.2	457	53.3	475	55.0	
	Low (0–3)	329	19.1	166	19.4	163	18.9	
**Child lifestyle factors**								
Breakfast eating	Every day	1600	93.0	805	93.9	795	92.0	0.120
	Skipping	121	7.0	52	6.1	69	8.0	
Sleep duration	More than 8 h	1358	78.9	685	79.9	673	77.9	0.300
	Less than 8 h	363	21.1	172	20.1	191	22.1	
Preference for physical activity	Like very much	1486	86.3	744	86.8	742	85.9	0.572
	Dislike	235	13.7	113	13.2	122	14.1	
Frequency of physical activity	Very often	1245	72.3	635	74.1	610	70.6	0.105
	Not often	476	27.7	222	25.9	254	29.4	
Children’s ST, h/day	< 2 h	1050	61.0	481	56.1	569	65.9	< 0.001
	≥ 2 h	671	39.0	376	43.9	295	34.1	
Cram schools	≥ 2 times/week	337	19.6	170	19.8	167	19.3	0.684
	1 times/week	91	5.3	49	5.7	42	4.9	
	0 time	1293	75.1	638	74.4	655	75.8	
**Children’s health**								
Overall health	Good	1598	92.9	799	93.2	799	92.5	0.543
	Poor	123	7.1	58	6.8	65	7.5	
**Enrichment of school life**								
Have close friends	Yes	1673	97.2	825	96.3	848	98.1	0.018
	No	48	2.8	32	3.7	16	1.9	
Understand the school study	Understand well	1400	81.3	686	80.0	714	82.6	0.167
	Not understand	321	18.7	171	20.0	150	17.4	

*IU* Internet use, *ST* Screen time

Table 2 shows the results of logistic regression analyses conducted to determine the strengths of the associations between “do not like physical activity” and social background, parental lifestyles, child lifestyles, overall child health, and enrichment of school life. The multivariate analysis revealed the following results: “do not like physical activity” for children in middle grades (3rd and 4th graders, ages 8–10) (adjusted OR, 2.39; 95% CI, 1.59–3.58), high grades (5th and 6th, ages 10–13) (adjusted OR 2.73; 95% CI, 1.83–4.08), poor communication with parents (adjusted OR 1.57; 95% CI, 1.10–2.25), long screen times of child (adjusted OR 1.38; 95% CI, 1.02–1.87), and do not have close friends (adjusted OR 5.30; 95% CI, 2.78–10.1). Among the boys’ main characteristics were long screen times of child and not attending cram school, and among the girls, poor communication with parents. Among the potential determinants of disliking physical activity, lack of close friends and grades were common factors for all participants.

**Table 2:** Logistic regression analysis results for “Don’t like physical activity” (*n* = 1721)

Variable		Total			Boys		Girls	
			Univariate	Multivariate		Multivariate		Multivariate
		%	OR (95% CI)	OR (95% CI)	%	OR (95%CI)	%	OR (95%CI)
Gender	Boys	13.2	1	1				
	Girls	14.1	1.08 (0.82–1.43)	1.25 (0.93–1.69)				
Grade	Low (1st–2nd)	7.1	1	1	7.2	1	7.1	1
	Middle (3rd–4th)	15.6	2.40 (1.62–3.56) ‡	2.39 (1.59–3.58) ‡	14.1	2.03 (1.13–3.64) *	17.0	2.77 (1.56–4.92) ‡
	High (5th–6th)	18.1	2.87 (1.97–4.20) ‡	2.73 (1.83–4.08) ‡	18.2	2.82 (1.59–5.01) ‡	18.0	2.80 (1.57–4.98) ‡
**Social background and parental lifestyles**								
Mother’s employment status	Full time	13.6	1	1	13.4	1	13.9	1
	Part time	13.0	0.95 (0.70–1.28)	0.91 (0.66–1.24)	12.7	0.86 (0.55–1.36)	13.3	0.97 (0.61–1.52)
	Unemployed (housewives)	15.9	1.20 (0.80–1.79)	1.33 (0.86–2.06)	14.2	1.11 (0.58–2.12)	17.6	1.52 (0.82–2.81)
Family structure	3-generation family	14.8	1	1	13.8	1	15.6	1
	Nuclear family	13.1	0.87 (0.65–1.16)	0.88 (0.65–1.19)	12.9	0.91 (0.58–1.43)	13.3	0.88 (0.58–1.34)
Perceived family affluence	Affluent	12.0	1	1	10.6	1	13.3	1
	Neither	13.4	1.14 (0.81–1.62)	1.11 (0.77–1.59)	12.7	1.24 (0.72–2.13)	14.2	1.09 (0.66–1.80)
	No affluent	15.8	1.38 (0.94–2.02)	1.21 (0.81–1.82)	16.5	1.54 (0.86–2.77)	15.0	0.98 (0.55–1.77)
Communication with parents	Often	12.3	1	1	12.5	1	12.2	1
	Rarely	20.1	1.79 (1.29–2.49) ‡	1.57 (1.10–2.25) *	15.5	1.04 (0.64–1.68)	31.4	2.87 (1.65–4.99) ‡
Father’s IU at home, h/day	< 2 h	13.7	1	1	13.2	1	14.1	1
	≥ 2 h	13.6	0.99 (0.67–1.47)	0.89 (0.58–1.36)	13.1	0.81 (0.44–1.50)	14.2	0.95 (0.52–1.73)
Mother’s IU at home, h/day	< 2 h	13.5	1	1	12.7	1	14.3	1
	≥ 2 h	16.2	1.23 (0.71–2.15)	1.02 (0.55–1.90)	20.0	1.40 (0.64–3.02)	10.3	0.51 (0.15–1.68)
Father’s behaviors based on Breslow’s health score	High (6, 7)	11.5	1	1	11.8	1	11.2	1
	Middle(4, 5)	13.1	1.16 (0.79–1.71)	1.04 (0.69–1.56)	12.8	0.99 (0.56–1.75)	13.4	1.09 (0.60–2.00)
	Low (0–3)	15.6	1.42 (0.96–2.09)	1.24 (0.81–1.91)	14.6	1.04 (0.57–1.93)	16.4	1.51 (0.81–2.80)
Mother’s behaviors based on Breslow’s health score	High (6, 7)	10.7	1	1	9.0	1	12.4	1
	Middle (4, 5)	14.7	1.45 (1.02–2.05) *	1.33 (0.92–1.92)	14.2	1.45 (0.84–2.53)	15.2	1.25 (0.75–2.10)
	Low (0–3)	14.9	1.47 (0.96–2.24)	1.25 (0.79–2.01)	16.3	1.74 (0.88–3.43)	13.5	0.98 (0.50–1.92)
**Child lifestyle factors**								
Breakfast eating	Every day	13.8	1	1	13.0	1	14.5	1
	Skipping	12.4	0.89 (0.51–1.55)	0.60 (0.33–1.07)	15.4	0.71 (0.30–1.64)	10.1	0.52 (0.22–1.21)
Sleep duration	More than 8 h	12.2	1	1	12.0	1	12.5	1
	Less than 8 h	19.0	1.69 (1.24–2.29) ‡	1.33(0.96–1.86)	18.0	1.21(0.73–1.99)	19.9	1.40 (0.88–2.23)
Children’s ST, h/day	< 2 h	11.4	1	1	10.0	1	12.7	1
	≥ 2 h	17.1	1.60 (1.22–2.11) ‡	1.38 (1.02–1.87) *	17.3	1.56 (1.00–2.44) *	16.9	1.21 (0.79–1.86)
Cram schools	≥ 2 times/week	11.6	1	1	8.2	1	14.4	1
	1 times/week	13.5	1.08 (0.53–2.21)	1.09 (0.52–2.27)	10.2	1.44 (0.47–4.44)	14.3	0.94 (0.34–2.60)
	0 time	13.6	1.32 (0.91–1.92)	1.24 (0.84–1.82)	14.7	1.86 (1.00–3.46) *	14.0	0.93 (0.56–1.55)
**Children’s health**								
Overall health	Good	13.1	1	1	12.8	1	13.4	1
	Poor	21.1	1.78 (1.13–2.81) *	1.32 (0.81–2.16)	19.0	1.03 (0.49–2.17)	23.1	1.62 (0.83–3.18)
**Enrichment of school life**								
Have close friends	Yes	12.8	1	1	12.0	1	13.6	1
	No	43.8	5.30 (2.95–9.55) ‡	5.30 (2.78–10.1) ‡	43.8	6.09 (2.73–13.6) ‡	43.8	4.37 (1.29–14.75) *
Understand the school study	Understand well	13.0	1	1	13.0	1	13.0	1
	Not understand	16.5	1.32 (0.95–1.85)	1.08 (0.76–1.54)	14.0	0.83 (0.49–1.41)	19.3	1.33 (0.81–2.18)

% Do not like physical activity

*OR* Odds ratio, *95% CI* 95% Confidence interval, *IU* Internet use, *ST* Screen time

*p < 0.05, ^†^*p* < 0.01, ^‡^*p* < 0.001

Table 3 shows the results of the logistic regression analyses conducted to determine the strengths of the associations between “lack of physical activity” and social background, parental lifestyles, child lifestyles, overall child health, and enrichment of school life. The multivariate analyses revealed the following associations with lack of physical activity: girls (adjusted OR 1.40; 95% CI, 1.11–1.76), middle grades (3rd and 4th graders, ages 8–10; adjusted OR 1.53; 95% CI, 1.15–2.04), high grades (5th and 6th graders, ages 10–13; adjusted OR 1.82; 95% CI, 1.37–2.41), unemployed mother (housewives; adjusted OR 1.91; 95% CI, 1.37–2.68), poor communication with parents (adjusted OR 1.59; 95% CI, 1.19–2.13), mothers with middle health behaviors (adjusted OR 1.50; 95% CI, 1.13–1.99), mothers with low health behaviors (adjusted OR 1.54; 95% CI, 1.07–2.20), long screen times of child (adjusted OR 1.47; 95% CI, 1.17–1.86), bad overall health (adjusted OR 1.66; 95% CI, 1.12–2.47), and lack of close friends (adjusted OR 5.40; 95% CI, 2.81–10.4). Among the boys’ associated characteristics were mothers with middle health behaviors, mothers with low health behaviors, and long screen times of child; and for girls’ characteristics, part-time-employed mother, unemployed mother (housewives), poor communication with parents, fathers with middle health behaviors, fathers with low health behaviors, long screen times of child, and bad overall health. Among the potential determinants of lack of physical activity, lack of close friends and long screen times were common factors for all participants.

**Table 3:** Logistic regression analysis results for “Lack of physical activity” (*n* = 1721)

Variable		Total			Boys		Girls	
			Univariate	Multivariate		Multivariate		Multivariate
		%	OR (95% CI)	OR (95% CI)	%	OR (95% CI)	%	OR (95% CI)
Gender	Boys	25.9	1	1				
	Girls	29.4	1.19 (0.96–1.47)	1.40 (1.11–1.76) ^†^				
Grade	Low (1st–2nd)	21.3	1	1	22.3	1	20.2	1
	Middle (3rd–4th)	28.8	1.50 (1.14–1.97) †	1.53 (1.15–2.04) †	25.3	1.12 (0.74–1.69)	32.2	2.16 (1.42–3.27) ‡
	High (5th–6th)	32.7	1.80 (1.39–2.34) ‡	1.82 (1.37–2.41) ‡	30.1	1.42 (0.94–2.13)	35.3	2.49 (1.65–3.78) ‡
**Social background and parental lifestyles**								
Mother’s employment status	Full time	24.5	1	1	25.2	1	23.8	1
	Part time	27.8	1.19 (0.94–1.50)	1.18 (0.92–1.50)	25.7	1.00 (0.70–1.43)	29.9	1.44 (1.01–2.06) *
	Unemployed (housewives)	36.2	1.75 (1.28–2.39) ‡	1.91 (1.37–2.68) ^‡^	28.3	1.17 (0.71–1.91)	44.5	3.38 (2.08–5.49) ‡
Family structure	3-generation family	28.0	1	1	24.3	1	31.3	1
	Nuclear family	27.5	0.97 (0.78–1.22)	0.95 (0.75–1.20)	26.7	1.13 (0.79–1.61)	28.4	0.79 (0.56–1.10)
Perceived family affluence	Affluent	25.9	1	1	26.0	1	25.8	1
	Neither	28.2	1.13 (0.87–1.46)	1.06 (0.81–1.39)	26.4	1.01 (0.68–1.49)	30.0	1.20 (0.81–1.79)
	No affluent	28.4	1.14 (0.85–1.53)	0.93 (0.68–1.27)	25.0	0.80 (0.51–1.26)	32.4	1.05 (0.66–1.66)
Communication with parents	Often	25.9	1	1	24.3	1	27.2	1
	Rarely	36.2	1.62 (1.24–2.11) ‡	1.59 (1.19–2.13) †	30.9	1.26 (0.87–1.82)	48.8	2.25 (1.36–3.71) †
Father’s IU at home, h/day	< 2 h	27.0	1	1	25.7	1	28.2	1
	≥ 2 h	31.6	1.25 (0.93–1.67)	1.06 (0.77–1.46)	26.9	0.88(0.55–1.41)	36.7	1.23 (0.78–1.94)
Mother’s IU at home, h/day	< 2 h	26.9	1	1	25.0	1	28.8	1
	≥ 2 h	39.4	1.76 (1.16–2.68) †	1.38 (0.86–2.21)	38.3	1.47 (0.79–2.71)	41.0	1.32 (0.63–2.80)
Father’s behaviors based on Breslow’s health score	High (6, 7)	22.7	1	1	26.2	1	19.1	1
	Middle (4, 5)	27.1	1.27 (0.94–1.70)	1.15 (0.84–1.57)	24.7	0.87 (0.57–1.34)	29.7	1.68 (1.04–2.71) *
	Low (0–3)	31.1	1.53 (1.14–2.07) †	1.28 (0.92–1.79)	27.2	0.85 (0.53–1.35)	34.5	2.11 (1.28–3.48) †
Mother’s behaviors based on Breslow’s health score	High (6, 7)	20.7	1	1	17.5	1	23.9	1
	Middle (4, 5)	29.7	1.62 (1.25–2.12) ‡	1.50 (1.13–1.99) †	27.1	1.68 (1.11–2.56) *	32.2	1.36 (0.91–2.03)
	Low (0–3)	31.6	1.78 (1.28–2.46) ‡	1.54 (1.07–2.20) *	34.3	2.53 (1.51–4.23) ‡	28.8	0.88 (0.52–1.49)
**Child lifestyle factors**								
Breakfast eating	Every day	27.6	1	1	25.6	1	30.8	1
	Skipping	28.9	1.07 (0.71–1.61)	0.74 (0.48–1.15)	30.8	0.91 (0.47–1.77)	27.5	0.66 (0.36–1.22)
Sleep duration	More than 8 h	26.8	1	1	25.5	1	27.3	1
	Less than 8 h	30.9	1.22 (0.95–1.57)	1.03 (0.78–1.36)	27.3	0.93 (0.61–1.41)	27.3	1.11 (0.76–1.63)
Children’s ST, h/day	< 2 h	23.4	1	1	21.0	1	25.5	1
	≥ 2 h	34.3	1.71 (1.38–2.11) ‡	1.47 (1.17–1.86) ‡	32.2	1.55 (1.10–2.17) *	36.9	1.47 (1.05–2.06) *
Cram schools	≥ 2 times/week	27.0	1	1	32.3	1	27.0	1
	1 times/week	23.1	0.81 (0.47–1.40)	0.78 (0.44–1.37)	26.2	0.95 (0.42–2.17)	23.1	0.68 (0.30–1.54)
	0 time	28.2	1.06 (0.81–1.39)	0.97 (0.73–1.28)	28.9	1.29 (0.84–1.97)	28.2	0.75 (0.51–1.12)
**Children’s health**								
Overall health	Good	26.5	1	1	25.0	1	28.0	1
	Poor	42.3	2.03 (1.39–2.95) ‡	1.66 (1.12–2.47) *	37.9	1.34 (0.74–2.43)	46.2	1.96 (1.11–3.45) *
**Enrichment of school life**								
Have close friends	Yes	26.5	1	1	24.5	1	28.5	1
	No	66.7	5.54 (3.01–10.2) ‡	5.40 (2.81–10.4) ‡	62.5	5.51(2.52–12.03) ‡	75.0	6.68 (1.86–23.93) †
Understand the school study	Understand well	26.9	1	1	25.8	1	27.9	1
	Not understand	31.2	1.23 (0.95–1.60)	1.02 (0.77–1.36)	26.3	0.86(0.57–1.29)	36.7	1.23 (0.82–1.84)

% Lack of physical activity

*OR* Odds ratio, *95% CI* 95% Confidence interval, *IU* Internet use, *ST* Screen time

*p < 0.05, †*p* < 0.01, ‡*p* < 0.001

While children who lacked physical activity comprised 27.7% of the total, more than half said that they like physical activity. However, many still did not engage in physical activity. In the analyses, we used the data of a total of 1486 participants (744 boys and 742 girls) who liked physical activity (Fig. 1). Table 4 shows the results of the logistic regression analysis conducted to determine the strengths of the associations between “lack of physical activity” regardless of “preference for physical activity” and social background, parental lifestyles, child lifestyles, overall child health, and enrichment of school life. The multivariate analyses revealed that girls (adjusted OR 1.52; 95% CI, 1.13–2.04), unemployed mothers (housewives; adjusted OR 2.30; 95% CI, 1.52–3.47), poor communication with parents (adjusted OR 1.50; 95% CI, 1.03–2.19), mothers with middle health behaviors (adjusted OR 1.55; 95% CI, 1.08–2.24), long screen times of child (adjusted OR 1.54; 95% CI, 1.15–2.08), and lack of close friends (OR 4.14; 95% CI, 1.80–9.51) were associated with “lack of physical activity” regardless of “preference for physical activity.” Among key characteristics, for boys they were non-affluence, and mothers with low health behaviors were key, while for girls they were middle grades, high grades, part-time-employed mothers, unemployed mothers (housewives), fathers with middle health behaviors, fathers with low health behaviors, and long screen times of child. Among the potential determinants of a lack of physical activity regardless of preference for physical activity, lack of close friends was a common factor for all participants.

**Table 4:** Logistic regression analysis results for “Lack of physical activity” regardless of “Prefer physical activity” (*n* = 1486)

Variable		Total			Boys		Girls	
			Univariate	Multivariate		Multivariate		Multivariate
		%	OR (95%CI)	OR (95%CI)	%	OR (95%CI)	%	OR (95%CI)
Gender	Boys	15.1	1	1				
	Girls	18.7	1.30 (0.99–1.71)	1.52 (1.13–2.04) †				
Grade	Low (1st–2nd)	15.4	1	1	16.2	1	14.5	1
	Middle (3rd–4th)	16.3	1.07 (0.76–1.51)	1.12 (0.78–1.59)	13.0	0.74 (0.44–1.25)	19.7	1.72 (1.03–2.87) *
	High (5th–6th)	19.1	1.30 (0.94–1.79)	1.33 (0.94–1.88)	15.7	0.86 (0.51–1.45)	22.3	2.09 (1.26–3.48) †
**Social background and parental lifestyles**								
Mother’s employment status	Full time	13.4	1	1	14.0	1	12.9	1
	Part time	17.5	1.36 (1.00–1.85) *	1.36 (0.99–1.87)	15.2	1.11 (0.70–1.78)	19.8	1.80 (1.14–2.86) *
	Unemployed(housewives)	25.1	2.16 (1.46–3.20) ‡	2.30 (1.52–3.47) ‡	17.4	1.28 (0.68–2.42)	33.7	4.58 (2.54–8.26) ‡
Family structure	3-generation family	16.1	1	1	12.1	1	12.1	1
	Nuclear family	17.3	1.09 (0.81–1.45)	1.01 (0.74–1.37)	16.4	1.29 (0.80–2.09)	16.4	0.76 (0.50–1.16)
Perceived family affluence	Affluent	16.8	1	1	17.7	1	15.8	1
	Neither	17.7	1.06 (0.77–1.47)	0.99 (0.71–1.39)	16.0	0.86 (0.53–1.40)	19.2	1.23 (0.74–2.03)
	No affluent	15.5	0.91 (0.62–1.34)	0.72 (0.48–1.08)	10.7	0.45 (0.24–0.83) *	21.0	1.04 (0.58–1.85)
Communication with parents	Often	16.1	1	1	14.1	1	17.9	1
	Rarely	20.9	1.38 (0.97–1.95)	1.50 (1.03–2.19) *	18.3	1.34 (0.83–2.17)	28.8	1.76 (0.92–3.37)
Father’s IU at home, h/day	< 2 h	16.1	1	1	14.9	1	17.4	1
	≥ 2 h	21.3	1.41 (0.98–2.01)	1.16 (0.78–1.71)	15.9	0.92 (0.50–1.67)	27.2	1.37 (0.79–2.37)
Mother’s IU at home, h/day	< 2 h	16.3	1	1	14.5	1	18.0	1
	≥ 2 h	27.7	1.98 (1.20–3.26) †	1.55 (0.89–2.69)	22.9	1.48 (0.67–3.23)	34.3	1.70 (0.74–3.92)
Father’s behaviors based on Breslow’s health score	High(6, 7)	13.3	1	1	17.0	1	9.5	1
	Middle(4, 5)	16.6	1.30 (0.89–1.91)	1.17 (0.78–1.75)	13.7	0.78 (0.45–1.33)	19.8	2.10 (1.10–4.00)
	Low (0–3)	19.4	1.56 (1.06–2.30) *	1.24 (0.81–1.89)	15.5	0.71 (0.39–1.29)	22.8	2.45 (1.26–4.77)
Mother’s behaviors based on Breslow’s health score	High (6, 7)	11.9	1	1	10.3	1	13.6	1
	Middle (4, 5)	18.4	1.66 (1.17–2.35) †	1.55 (1.08–2.24) *	15.3	1.69 (0.98–2.93)	21.3	1.45 (0.87–2.41)
	Low (0–3)	20.0	1.85 (1.22–2.81) †	1.57 (0.99–2.49)	21.6	2.81 (1.44–5.47) †	18.4	0.82 (0.42–1.60)
**Child lifestyle factors**								
Breakfast eating	Every day	16.7	1	1	14.7	1	18.7	1
	Skipping	19.8	1.24 (0.75–2.03)	1.01 (0.60–1.71)	20.5	1.46 (0.64–3.35)	19.4	0.83 (0.40–1.72)
Sleep duration	More than 8 h	17.1	1	1	15.6	1	18.7	1
	Less than 8 h	16.0	0.92 (0.65–1.30)	0.86 (0.59–1.24)	12.8	0.75 (0.42–1.35)	19.0	0.96 (0.58–1.59)
Children’s ST, h/day	< 2 h	14.0	1	1	12.5	1	15.3	1
	≥ 2 h	21.8	1.71 (1.30–2.25) ‡	1.54 (1.15–2.08) †	18.6	1.50 (0.96–2.35)	25.7	1.76 (1.16–2.66) *
Cram schools	≥ 2 times/week	18.4	1	1	15.4	1	21.7	1
	1 times/week	13.8	0.79 (0.41–1.51)	0.65 (0.32–1.34)	11.4	0.67 (0.23–1.97)	16.7	0.67 (0.24–1.86)
	0 time	16.7	0.96 (0.70–1.32)	0.80 (0.57–1.14)	15.3	0.95 (0.57–1.60)	18.1	0.69 (0.43–1.13)
**Children’s health**								
Overall health	Good	16.2	1	1	14.5	1	17.9	1
	Poor	26.8	1.89 (1.18–3.04) †	1.63 (1.00–2.68)	23.4	1.51 (0.71–3.20)	30.0	1.53 (0.76–3.10)
**Enrichment of school life**								
Have close friends	Yes	16.4	1	1	14.6	1	18.3	1
	No	40.7	3.49 (1.60–7.62) †	4.14 (1.80–9.51) ‡	33.3	4.05 (1.37–11.9) †	55.6	6.14 (1.45–25.97) *
Understand the school study	Understand well	16.7	1	1	15.2	1	18.0	1
	Not understand	17.9	1.09 (0.77–1.54)	0.91 (0.63–1.32)	14.3	0.81 (0.47–1.39)	22.3	0.96 (0.57–1.62)

% Lack of physical activity

*OR* Odds ratio, *95% CI* 95% Confidence interval, *IU* Internet use, *ST* Screen time

*p < 0.05, †*p* < 0.01, ‡*p* < 0.001

We performed logistic regression analyses by stratified grade level (low: 1st and 2nd grades, middle: 3rd and 4th grades, high: 5th and 6th grades) and assessed the Hosmer−Lemeshow statistic, and the regression equation was judged to be invalid (data not shown). Table 5 shows the results of “have close friends,” analyzed by stratified grade. According to the results of a Chi-squared test, there was no significant differences in “have close friends” according to grade level.

**Table 5:** Characteristics of “Have close friends” analyzed by stratified grade (*p* = 0.786)

Grade	Yes		No		Total
	(*n*)	(%)	(*n*)	(%)	(*n*)
Low (1st–2nd)	557	97.0	17	3.0	574
Middle (3rd–4th)	532	97.6	13	2.4	545
High (5th–6th)	584	97.0	18	3.0	602

Family structure, father’s IU at home in h/day, and breakfast consumption were not found to be significant in the univariate and multivariate analyses. The correlation coefficients between independent variables ranged from 0.000 to 0.323, indicating no multicollinearity. The Hosmer−Lemeshow tests validated the models.

## Discussion

Among the potential determinants of child exercise habits, this study revealed that a lack of close friends was likely the most influential factor for both disliking and lacking physical activity. Further, having close friends was independently associated with child exercise habits even after adjusting for other potential confounding factors, including the child’s social and family environment and child lifestyle factors.

Previous studies have shown that peer support is important for promoting exercise habits [7], while the presence of friends promotes activeness [30] and reduces time spent sitting [31]. Our findings showed that children with screen times of 2 or more hours per day were more likely to dislike and lack physical activity when compared to those with screen times of less than 2 h. Internet addiction and gaming disorders are both recognized by the World Health Organization [32]; recent surveys have also highlighted that these disorders especially affect young children, that it is more difficult for children than for adults to recover, and that experiences related to physical activity and increased peer communication are effective for prevention and treatment [33]. Offline interactions between children are becoming scarcer; as such, approaches to help them make friends and form childhood relationships are an essential educational goal. Schoolchildren evaluated these education models positively, and their consciousness about social behaviors changed positively [34]; however, according to a 2015 survey, compared to 1995, the number of friends with whom children play after school had decreased, the number of children who played with their mothers instead of friends increased [35]. These data suggest that the environment surrounding children is changing, and the opportunities for children to engage in physical activity with friends after school are decreasing. Since it has been reported that children’s peer relationships are enriched and expanded between the ages of 6−12 years [36], the definition of “having close friends” could vary for children of different ages. However, this study found no significant differences for “have close friends” according to grade level. Thus, it might be beneficial to have close friends from preschool years in the promotion of physical activity, although longitudinal research is necessary.

Family structure, father’s IU at home in h/day, and breakfast consumption were not significant in the univariate and multivariate analyses. This may be due to the difference between children with and without habits being very small. This study found family-related factors that influenced dislike and lack of physical activity, including having housewives, unhealthy parental lifestyles, and lack of communication with parents. Children in these groups were more often sedentary, even if they reported that they liked physical activity. This study also found that childhood exercise habits may be influenced by parent lifestyles and that children may be deficient in physical activity due to social and family factors even when they like physical activity. Previous studies have shown that families lacking in parent–child communication were more sedentary and had lower health satisfaction [22]; conversely, children who frequently talked with their parents had better bowel movements, indicating proper digestion [24]. Further, children develop an attachment to their parents through physical touch and communication [37], and healthy parent–child communication and healthy parental lifestyles are essential for childhood mental and physical health, as well as children’s development of healthy exercise habits.

This study found cross-gender associations (between father−daughter and mother−son pairs) between parental unhealthy lifestyles (as assessed using Breslow’s seven health-related behaviors) and lack of physical activity. It has been reported that mothers’ unhealthy lifestyles, as assessed using Breslow’s seven health-related behaviors, are associated with prolonged screen time in children [10]. Other studies using similar indicators have shown that children whose mothers have unhealthy lifestyles are more likely to be sedentary [22]. This is considered to reflect the important role mothers have in raising children in Japanese society [9]. However, children whose fathers have strong preferences for exercise have been shown to receive better exercise support than children whose fathers have no preference for exercise [38]. There were significant correlations found between physical activity in daughters and exercise support (e.g., parent–child co-activity, praising the child for being active) received from fathers [39]. Although further research is needed to understand the underlying mechanisms, fathers’ lifestyle factors (especially exercise habits) may influence their children’s engagement in physical activity.

Previous studies [9] have also reported that longer screen times, later bedtimes, and poorer lifestyle habits were more common among children with mothers who worked full-time. Conversely, more recent studies have shown that children with mothers who worked full-time have physical activity times, thus suggesting they were less affected by maternal employment status [24]. This study’s results further support the idea that maternal employment status and/or the way children spend their after-school hours have changed over time. For example, after-school clubs and services are now available [40] for children whose parents or legal guardians are away from home during the initial after-school hours. This is partially the result of the Act on the Promotion of Female Participation and Career Advancement in the Workplace, which was passed in August 2015. Further, a 2017 survey [41] indicated that such after-school clubs provide space for and promote physical activity. It is therefore possible that children with working mothers are becoming more able to engage in physical activity outside and with friends.

The UNICEF Report Card on Child Well-being in a Sustainable World [42] also suggested that family was the most important factor for childhood well-being, followed by friends and school. Perhaps the importance of close friends and family communication for developing good exercising habits, as found in our study, reflects the “wellness” that children in developed countries experience. Healthy exercise habits in childhood are most effectively promoted through experiencing a fulfilling school life; however, a child’s home life is also important. Activities after school and on weekends can be improved by promoting shorter screen times, better parental lifestyles, and improved parent–child communication. Previous research has shown significant correlations between children’s physical activity levels and parental support [38] or encouragement [39], suggesting the importance of family-based shared activity interventions for child development [39]. Thus, to increase children’s physical activity levels and create positive exercise habits, parental cooperation is necessary when developing strategies for such interventions.

## Limitations

This study has some limitations that should be noted. First, previous studies have noted that poor childhood exercise habits are common in low-income families [43]. In this study, although there was a significant difference found among boys, it was not very strong and varied depending on the number of participants. In a previous study using the same survey items, children’s longer screen time [10] and poor dietary habits [11] were associated with low family income. The survey used in this study was tailored toward the respondents and may not be able to grasp the economic situation; thus, further research is needed. Second, this study used a cross-sectional design that employed a self-report survey on exercise habits. Thus, a longitudinal survey is needed to investigate the causal relationships underlying these results, including the relationship between children’s physical activity and close friendship, and measure both the quality and quantity of children’s physical activity through objective indicators. Third, more detailed surveys on social context (e.g., community characteristics, maternal employment status, and social assistance) are required to investigate the measures best suited for community-specific needs.

## Conclusions

This study investigated factors related to both dislike and lack of physical activity among schoolchildren as part of the Super Shokuiku School Project in Japan. “Lack of close friends” had the strongest links with both dislike and lack of physical activity. Children who engaged in long periods of screen time and lacked parental communication also tended to dislike and lack physical activity. Although a longitudinal study is needed to determine causality, substantial attention to children’s lifestyle behaviors after school may thus be required to promote physical activity. Further, to increase children’s engagement in physical activity, parental cooperation is warranted in intervention strategies.

## Data Availability

The data we used to derive our findings are unsuitable for public deposition due to ethical restrictions and specific legal framework in Japan. It is prohibited by the Act on the Protection of Personal Information (Act No.57 of 30 May 2003, amended on 9 September 2015) to publicly deposit data containing personal information. The Ethical Guidelines for Epidemiological Research enforced by the Japan Ministry of Education, Culture, Sports, Science, and Technology and the Ministry of health, Labor and Welfare also restrict the open sharing of the epidemiologic data.
